# Investigation of Health-Related Quality of Life, Anxiety and Satisfaction in Patients with Pulmonary Embolism

**DOI:** 10.3390/jpm14040393

**Published:** 2024-04-08

**Authors:** Foteini Malli, Niki Gkena, Diamantoula Papamichali, Nikoletta Vlaikoudi, Ioanna V. Papathanasiou, Evangelos C. Fradelos, Dimitrios Papagiannis, Erasmia C. Rouka, Dimitrios G. Raptis, Zoe Daniil, Konstantinos I. Gourgoulianis

**Affiliations:** 1 Respiratory Medicine Department, Faculty of Medicine, University of Thessaly, Biopolis, 41110 Larissa, Greece; raptisdmed@gmail.com (D.G.R.); zdaniil@uth.gr (Z.D.); kgourg@med.uth.gr (K.I.G.); 2 Respiratory Disorders Lab, Faculty of Nursing, School of Health Sciences, University of Thessaly, 41110 Larissa, Greece; gkenaniki@gmail.com (N.G.); papa2@uth.gr (D.P.); nivlaikoudi@uth.gr (N.V.); 3 Faculty of Nursing, School of Health Sciences, University of Thessaly, 41110 Larissa, Greece; iopapathanasiou@uth.gr (I.V.P.); efradelos@uth.gr (E.C.F.); rrouka@uth.gr (E.C.R.); 4 Public Health & Vaccines Lab, Faculty of Nursing, School of Health Sciences, University of Thessaly, 41110 Larissa, Greece; dpapajohn@uth.gr

**Keywords:** anxiety, pulmonary embolism, quality of life, satisfaction with life, thrombosis, thromboembolism

## Abstract

Background: Features of post-traumatic stress disorder and anxiety may be present in pulmonary embolism (PE) patients, along with impaired quality of life (QoL). We aim to evaluate health-related QoL, anxiety and satisfaction with life in patients with PE. Methods: Patients with PE were enrolled during their follow-up. All participants completed the Short Form 36 (SF-36) questionnaire, the State–Trait Anxiety Inventory (STAI) X1 and X2 forms, and the Satisfaction with Life Scale (SWLS). Results: 92 PE patients were included (mean age ± SD = 62.50 ± 15.33 years, 56.5% males). The median values of the SF-36 subscales were below the corresponding values of the Greek general population (besides the mental health (MH) subscale). Mean STAIX1 levels were 37.05 ± 11.17 and mean STAIX2 levels were 39.80 ± 10.47. Mean SWLS levels were 23.31 ± 6.58. According to multiple linear regression analysis, the MH and general health subscales were predictive of SWLS levels (F (10.76) = 10.576, *p* < 0.001, R2 = 0.581). The MH score (β = −0.242, *p* < 0.01) and STAIX1 level (β = 0.312, *p* < 0.001) (F (9.77) = 26.445, *p* < 0.001, R2 = 0.756) were predictive of STAIX2. Conclusions: Patients with PE exhibit slight satisfaction with life, borderline anxiety and impaired HRQoL.

## 1. Introduction

Venous thromboembolism (VTE) is a common disorder that includes pulmonary embolism (PE) and deep vein thrombosis (DVT) [1,2]. PE is the most serious manifestation of VTE, accounting for approximately 300,000 deaths annually in USA [3]. With timely treatment, many of the survivors fully recover within months [1]. However, some of them may experience long-term sequelae, such as chronic thromboembolic disease. Interestingly, the long-term psychological consequences of PE are less well understood and scarcely studied in the literature.

Changes in health-related (HR) QoL is an important outcome in several medical conditions and is related to the presence of chronic disease and/or associated risk factors; impaired HRQoL is closely linked to mortality in both people with chronic diseases and the general population [4,5]. The definition of HRQoL encompasses the patients’ self-reported impact of a disease and its associated treatment on his/her physical, mental and social functioning and wellbeing [6]. Satisfaction with life is a broader component of subjective wellbeing through which individuals address the quality of their lives in terms of their own criteria that depend on their personal priorities of life [7]. Life satisfaction can be used as a promising tool for the assessment of positive psychological attributes, which may be linked to changes in mortality and life expectancy [8]. Trait anxiety refers to the persons’ tendency to present state anxiety. State anxiety mirrors the psychological and physical reactions directly related to demanding situations in a specific moment [9]. The measurement of and distinction between those two aspects is mandatory for the assessment and follow-up of an individual’s anxiety levels, and it may help to effectively manage anxiety [10].

The impact of PE may extend beyond the physical consequences of the disease. PE can be considered a health-related crisis for various reasons, including the experience of an acute and life-threatening diagnosis, with potentially invasive treatment, the long-term use of anticoagulants, the fear of recurrences, chronic complications and/or adverse events due to anticoagulant use [11]. Features of post-traumatic stress disorder and anxiety may be present in some PE patients [12], while impaired QoL may be found in most of them following an acute event [13]. The understanding of the effect of PE recovery in relation to psychological and mental wellbeing merits research.

Since the year 2019, when it was first reported, severe acute respiratory syndrome coronavirus 2 (SARS-CoV-2), which causes coronavirus disease 2019 (COVID-19), has been considered one of the greatest global pandemics in modern history [14]. Despite governmental restrictions, national lockdowns and safety measures, in Greece the virus spread, leading to hospital admissions and deaths [15]. PE is one severe complication associated with COVID-19 [16], and a pre-existing PE in a patient may complicate the COVID-19 infection [17]. It has been reported that many patients suffering from various diseases have modified their health visits during the global pandemic and experienced psychological distress and poor QoL [18,19,20].

We hypothesized that patients with PE may present impaired HRQoL, which may be associated with anxiety and life satisfaction. We chose anxiety and satisfaction with life since they both present important aspects of mental health in the general population but have not been examined previously (satisfaction with life) or have not been examined thoroughly (anxiety) in the PE population [21]. To this end, we aimed to evaluate HRQoL, anxiety and satisfaction with life in PE patients. Additionally, we sought to address possible correlations between the aforementioned mental health aspects.

## 2. Materials and Methods

Patients with PE, with or without DVT, were enrolled consecutively between April 2020 and June 2021 during their follow-up at the PE Outpatient Clinic of the Respiratory Medicine Department of the University Hospital of Larissa, Greece. The inclusion criteria included a PE diagnosis at least 1 month prior to enrollment in the study. All participants had their PE confirmed through Computed Tomography Pulmonary Angiography (CTPA). Concurrent DVT was diagnosed with whole-leg compression ultrasonography. Exclusion criteria included age < 18 years, dementia and refusal to provide written consent. Demographic and clinical characteristics of the patients were recorded. Unprovoked PE was defined as PE occurring in the absence of transient or persistent major or intermediate risk factors [1,22]. Acute right heart dysfunction was diagnosed according to current guidelines [1]. High-risk PE was defined as PE occurring in the context of hemodynamic instability [1]. All participants completed the SF-36 questionnaire, the State–Trait Anxiety Inventory (STAI) form X-1 (STAIX1) and form X-2 (STAIX2) and the Satisfaction with Life Scale (SWLS). We used the Short Form 36 (SF-36) questionnaire to address HRQoL. SF-36 provides an accurate estimate of the impact of illnesses on a patient’s quality of life [23] and is considered the most extensively validated measure of QoL in various populations [24]. The SF-36 questionnaire is validated in the Greek language [25]. SF-36 consists of eight subscales: vitality (VT), physical functioning (PF), bodily pain (BP), general health perceptions (GH), physical role functioning (RP), emotional role functioning (RE), social role functioning (SF) and mental health (MH). Each subscale is rated between 0 and 100, with higher scores indicating better health status. A mean score of 50 is considered a normative value for all subscales [13,26].

We used the STAIX1 and X2 forms to assess state and trait anxiety, respectively. The instruments present high inner validity for the clinical diagnosis [27]. Trait anxiety was assessed using STAIX2, which refers to anxiety as a personality trait [28]. STAIX2 includes questions relating to how one feels generally. State anxiety was assessed using STAIX1, which refers to anxiety as a condition. STAIX1 asks respondents how they currently feel, STAIX2 how they generally feel. Scores range from 20 to 80. Higher scores suggest higher trait or state anxiety. Clinically significant anxiety corresponds to scores over 39 [29]. The STAI questionnaire is validated in the Greek language [30].

Finally, we used the SWLS, which measures subjective cognitive judgments of satisfaction with one’s life and is considered a valid and reliable instrument in various populations [7]. The SWLS is a 5-item instrument designed to measure global cognitive judgments of satisfaction with one’s life [31,32]. The total score ranges from 5 to 35. Subjects with higher scores present higher satisfaction with life. Scores between 5 and 9 suggest that the respondent is “extremely dissatisfied” with life, scores between 10 and 14 indicate the respondent is “dissatisfied”, scores between 15 and 19 suggest the respondent is “slightly dissatisfied”, a score of 20 is considered “neutral”, a score between 21 and 25 indicates the respondent is “slightly satisfied”, a score of 26–30 suggests the respondent is “satisfied”, whereas scores between 31–35 indicate the respondent is “extremely satisfied” [31].

### Statistical Analysis

Data are presented as mean ± SD or as percentages. Normal distribution was assessed using the Kolmogorov-Smirnov test. Univariate correlations were performed using Pearson’s correlation coefficient or Spearman’s correlation coefficient according to the variable’s distribution. Either the chi-squared test, Fischer’s exact test or Student’s t-test was used for the univariate analysis. The results of the SF-36 subscales were categorized as “below average” when the value was ≤50 and “over average” when the value was >50, since a cut-off of 50 is considered a normative value for all subscales [13,26]. For STAIX1 and STAIX2, we used a cut-off of 39 and categorized participants as having a high score if the value was >39 and a low score if the value was ≤39 [29]. We compared the results of the SF-36 subscales with the Greek healthy population norms [25] using the one-sample Wilcoxon rank test. We performed multiple linear regression analysis with SF-36 subscales, STAIX1, STAIX2 and SWLS scores as the dependent variables. Variables with a significance level below 0.05 were retained for the analysis and served as possible independent predictors. The coefficient of determination (R squared, R^2^) was used to estimate the percentage of an effect explained by the model. The questionnaire’s reliability was assessed in terms of the Cronbach alpha coefficient. Internal consistency is considered acceptable when the Cronbach alpha coefficient is above 0.7 [32]. The significance level was set at *p* < 0.05. The sample size was calculated to provide a power of 80% (a = 0.05 based on a two-sided test), with a 10% margin error. For our sample (92 subjects), the margin of error was 2.84 (https://www.enterprise-development.org/measuring-results-the-dced-standard/sample-size-calculator/, accessed on 30 December 2022). Analysis was performed using the SPSS 20 statistical package (SPSS Chicago, IL, USA).

## 3. Results

The study included 92 PE patients. The patients’ sociodemographic characteristics are summarized in Table 1. The mean age (±SD) was 62.50 ± 15.33 years, and 56.5% were male. The mean disease duration was 9.58 ± 12.43 months. Most patients (52.2%) reported dyspnea at diagnosis, 8.7% of subjects had a history of recurrence, 43.5% had unprovoked VTE, 92.4% were on direct oral anticoagulant therapy and 8.7% reported minor hemorrhagic complications. Right heart dysfunction was evident in the acute event in 23.9%, and 8.7% experienced high-risk PE. Of the patients included, 8.7% had previous diagnosis of mental disease (depression, anxiety disorder). Hereditary thrombophilia (FV Leiden heterozygous or prothrombin G20210A heterozygous) was present in 7 out of the 61 patients examined. PE occurring in the setting of COVID-19 was observed in 4.4% of the study population (Table 1).

### 3.1. Health-Related Quality of Life, Anxiety and Life Satisfaction

Table 2 presents the SF-36, STAIX1, STAIX2 and SWLS scores. The Cronbach alpha value was above 0.8 in all subscales of the applied questionnaire (Supplementary Table S1). Most of the mean values of the SF-36 subscales were above the normative value, except for PR (45.92 ± 42.41). RE (51.26 ± 43.31) and GH (54.02 ± 18.79) were slightly above the normative value of 50. We compared the results from the SF-36 subscales with the corresponding norms of the Greek healthy population [25] (Table 2). We observed statistically significant differences in the values of all SF-36 subscales besides MH (Table 2), indicating an impaired HRQoL in our population.

The mean STAIX1 score was 37.05 ± 11.17 and the mean STAIX2 score was 39.80 ± 10.47. The mean SWLS score was 23.31 ± 6.58.

### 3.2. Bivariate Correlations

Table 3 presents the bivariate correlations of the SF-36 subscale, STAIX1, STAIX2 and SWLS scores and the demographic variables of the sample. Age of diagnosis was statistically significantly negatively correlated with PF, RP, VT, SF and GH (Table 3). Disease duration was statistically significantly positively correlated with RP, RE, SF and change in health (Table 3). BMI was statistically significantly negatively correlated with PF, VT and BP (Table 3).

We observed significant correlations between the SF-36 subscales (Table 4). PF was statistically significantly correlated with RP, RE, VT, MH, SF, BP and GH (Table 4). RP was significantly positively correlated with RE, VT, MH, SF, BP, GH and change in health (Table 4). RE was statistically significantly positively correlated with VT, MH, SF, BP and GH, and statistically significantly negatively correlated with change in health (Table 4). VT was statistically significantly positively correlated with MH, SF, BP, GH and change in health (Table 4). MH was statistically significantly positively correlated with SF, GH and change in health (Table 4). SF was statistically significantly positively correlated with BP (Table 4).

We observed significant correlations between the SF-36 subscale, STAI and SWLS results, which are presented in Table 4. STAIX1 was statistically significantly negatively correlated with PF, RP, RE, VT, MH, SF and GH (Table 4). STAIX2 was statistically significantly negatively correlated with PF, RP, RE, VT, MH, SF and GH (Table 4). SWLS was statistically significantly positively correlated with RP, RE, VT, MH, SF, BP and GH, and statistically significantly negatively correlated with PF (Table 4). STAIX1 was statistically significantly positively correlated with STAIX2 and negatively correlated with SWLS. STAIX2 was statistically significantly negatively correlated with SWLS (Table 4).

### 3.3. Comparison between Patient Characteristics and Psychometric Scores

#### 3.3.1. Demographics

We observed that men had higher levels of PF versus women (63.55 ± 24.66 vs. 50.25 ± 25.51, respectively, *p* = 0.013) (Supplementary Figure S1).

Former smokers exhibited the lowest SWLS scores (19.15 ± 8.56), followed by non-smokers (23.28 ± 6.49) and current smokers (24.81 ± 5.33) (*p* = 0.027).

Increased age of diagnosis was associated with worse HRQoL, as presented in the following sentences. Patients with below-average levels of PF had increased age of diagnosis (67.74 ± 14.12 years) versus those with over-average scores (57.20 ± 15.09 years) (*p* = 0.001). Patients with below-average RP had increased age of diagnosis (65.5 ± 13.79 years) versus those with over-average RP (55.89 + 16.20 years) (*p* = 0.004). Patients with below-average GH differed significantly to those with over-average GH in terms of age of diagnosis (65.97 ± 14.74 vs. 57.89 ± 15.25 years, respectively, *p* = 0.012). Patients with below-average VT had higher age of diagnosis (67.34 ± 12.34 years) when compared to those with over-average VT (58.19 ± 16.25 years) (*p* = 0.003). Patients with over-average BP had an increased age of diagnosis (67.61 ± 12.23 years) when compared to those with below-average BP (60.22 ± 15.90 years) (*p* = 0.039). Similarly, patients with below-average SF versus those with over-average scores had increased age of diagnosis (63.37 ± 10.31 vs. 57.28 ± 17.34 years, respectively, *p* = 0.001) and reduced disease duration (5.88 ± 6.46 vs. 12.48 ± 15.02 months, respectively, *p* = 0.007). Similarly, disease duration was reduced in those with below-average RP (6.91 ± 7.36 months) versus those with over-average RP (12.17 ± 17.14 months) (*p* = 0.046), indicating improvement in HRQoL over time.

#### 3.3.2. Clinical Characteristics of PE

Patients presenting dyspnea at diagnosis had higher levels of GH versus those without (58.12 ± 18.26 vs. 49.54.18.54, respectively, *p* = 0.028) (Supplementary Figure S1). Patients presenting hemoptysis at diagnosis had higher levels of BP than those without (91.25 ± 10.45 vs. 71.13 ± 28.82, respectively, *p* = 0.003) (Supplementary Figure S1). BP was lower in patients with prior VTE recurrence (50.00 ± 36.47) versus those without (74.58 ± 26.82) (*p* = 0.037) (Supplementary Figure S1). PF was higher in patients receiving anticoagulants than in patients that were not under anticoagulant therapy (59.52 ± 26.65 vs. 36.42 ± 16.76, respectively, *p* = 0.022) (Supplementary Figure S1). Patients who had high-risk PE more often had an over-average PF score (8.69%) than below-average PF (0%). Patients with PE associated with COVID-19 had lower change-in-health scores versus those whose events were not associated with SARS-CoV-2 (25.00 ± 20.41 vs. 59.77 ± 30.34, respectively, *p* = 0.026) (Supplementary Figure S1). Finally, patients with hereditary thrombophilia, versus those without, had higher levels of PF (82.14 ± 25.95 vs., 60.83 ± 24.52, respectively, *p* = 0.036), higher RE (95.23 ± 12.60 vs. 50.30 ± 43.97, respectively, *p* < 0.001), higher SF (98.21 ± 4.72 vs. 63.19 ± 28.95, respectively, *p* < 0.001) and higher BP (94.28 ± 9.75 vs. 72.03 ± 27.87, respectively, *p* < 0.001).

#### 3.3.3. Comorbidities

Patients with at least one comorbidity, versus those without, had lower SWLS scores (22.51 ± 6.81 vs. 27.26 ± 3.19, respectively, *p* < 0.001), lower SF (58.88 ± 32.26 vs. 76.56 ± 23.21, respectively, *p* = 0.041) and reduced GH (51.90 ± 17.98 vs. 64.06 ± 19.93, respectively, *p* = 0.018) (Supplementary Figure S2). Patients with diabetes mellitus, versus those without, had lower levels of RE (23.32 ± 35.30 vs. 54.67 ± 43.13, respectively, *p* = 0.023), lower MH (51.60 ± 21.74 vs. 68.54 ± 17.93, respectively, *p* = 0.009), lower GH (42.50 ± 18.59 vs. 55.42 ± 18.54, respectively, *p* = 0.039), higher STAIX1 scores (44.50 ± 15.46 vs. 36.12 ± 10.27, respectively, *p* = 0.025) and lower SWLS scores (18.30 ± 7.61 vs. 23.94 ± 6.21, respectively, *p* = 0.010). Patients with thyroid disease, when compared to those without, had lower PF (43.07 ± 23.14 vs. 60.18 ± 25.50, respectively, *p* = 0.026) and lower BP (54.03 ± 25.50 vs. 75.47 ± 27.85, respectively, *p* = 0.013). Patients with comorbidities had high SWLS scores more frequently (51.08%) than those without (16.30%) (*p* = 0.005).

Patients with known mental disease, versus those without, had increased STAIX1 scores (45.53 ± 9.65 vs. 35.36 ± 10.72, respectively, *p* = 0.001), increased STAIX2 scores (50.33 ± 10.16 vs. 37.69 ± 9.23, respectively, *p* < 0.001), reduced SWLS scores (17.60 ± 6.08 vs. 24.47 ± 6.09, respectively, *p* < 0.001), reduced VT (42.50 ± 17.88 vs. 6.02 ± 20.73, respectively, *p* = 0.002), lower MH (50.0 ± 18.81 vs. 70.22 ± 17.83, respectively, *p* < 0.001) and reduced GH (38.75 ± 9.21 vs. 57.23 ± 18.75, respectively, *p* < 0.001). Patients with known mental disease rarely had over-average VT (5.43%) compared to those without mental disease (11.95%) (*p* = 0.009). Patients with mental disease had below-average MH more frequently (6.52%) than over-average MH (10.86%), and those without mental disease more frequently had over-average MH (73.91%) than below-average MH (8.69%) (*p* = 0.014). Patients with over-average GH suffered more rarely from mental disease (1.08%) than those with below-average GH (16.30%) (*p* < 0.001). Patients with known mental disease more frequently had high STAIX1 levels than low levels (11.95% vs. 4.34%, respectively, *p* = 0.001). Similarly, patients with known mental disease more frequently had high STAIX2 levels (15.21%) than low levels (1.08%) (*p* < 0.001). Patients with known mental disease had high SWLS scores less frequently (5.43%) than low SWLS scores (61.95%) (*p* = 0.001).

### 3.4. Predictors of HRQoL, Anxiety and Satisfaction with Life

We performed multiple linear regression analysis to assess predictors of HRQoL, anxiety and satisfaction with life; the results are summarized in Table 5. In the multiple linear regression analysis, age (β = −0.469, *p* = 0.027) and BMI (β = −1.988, *p* = 0.001) significantly predicted PF score (F (12,32) = 4.623, *p* < 0.001, R2 = 0.634) (Supplementary Table S2). SF score (β = 0.457, *p* = 0.006), RE score (β = 0.404, *p* < 0.001) and PF score (β = 0.460, *p* = 0.014) were predictive of RP (F (13,70) = 7.749, *p* < 0.001, R2 = 0.590). Analysis for the prediction of RE demonstrated that only RP score (β = 0.493, *p* < 0.001) was predictive (F (12,71) = 6.295, *p* < 0.001, R2 = 0.515).

For VT score prediction, MH score (β = 0.715, *p* = 0.001) and GH score (β = 0.361, *p* = 0.017) served as independent variables (F (13,31) = 9.424, *p* < 0.001, R2 = 0.798). We observed that GH score (β = −0.235, *p* = 0.008), VT score (β = 0.296, *p* = 0.011), STAIX2 score (β = −0.972, *p* < 0.001) and SWLS score (β = 0.642, *p* = 0.019) were predictive of MH score (F (10,76) = 20.182, *p* < 0.001, R2 = 0.726). Only PR score (β = 0.229 *p* = 0.006) served as an independent variable for SF score (F (10,76) = 11.015, *p* < 0.001, R2 = 0.611).

In multiple linear regression analysis, only PF score (β = 0.413, *p* = 0.024) was significantly predictive of BP (F (10,81) = 4.904, *p* < 0.001, R2 = 0.546). For GH score, age (β = −0.236, *p* = 0.043) and SWLS score (β = 0.811, *p* = 0.019) were independent variables (F (10,76) = 8.242, *p* < 0.001, R2 = 0.520). Finally, for change-in-health score, only disease duration (β = 0.801, *p* = 0.011) served as an independent variable (F (6,82) = 3.457, *p* < 0.001, R2 = 0.202).

In the multiple linear regression model, only MH (β = 0.113, *p* = 0.015) and GH (β = 0.105, *p* = 0.00) were independent variables for the prediction of SWLS score (Table 4) (F (10,76) = 10.576, *p* < 0.001, R2 = 0.581). For STAIX1 score, only STAIX2 score served as an independent variable (β = 0.549, *p* < 0.001) (F (9,77) = 14.915, *p* < 0.001, R2 = 0.635). The following variables were independently predictive of STAIX2 score: MH score (β = −0.242, *p* < 0.01) and STAIX1 score (β = 0.312, *p* < 0.001) (F (9,77) = 26.445, *p* < 0.001, R2 = 0.756).

## 4. Discussion

In the present study, we examined for the first time a broad spectrum of mental health components (HRQoL, anxiety and life satisfaction) in a cohort of PE patients. Our patients presented reduced HRQoL when compared to the Greek general population. Age was inversely correlated with several factors of HRQoL and served as an independent variable for the prediction of PF and GH. Increased disease duration displayed positive correlations with some SF-36 subscales and was a significant variable for the prediction of change in health, suggesting that HRQoL may improve over time. Additionally, we found slightly increased trait anxiety and slight satisfaction with life. Anxiety was associated with impaired HRQoL, while satisfaction with life was associated with preserved HRQoL.

Life satisfaction has emerged as a distinct construct that reflects a cognitive evaluation of one’s life [33] and a potential target to improve one’s health and the use of preventive strategies [34]. Reduced life satisfaction has been associated with lower mortality and life expectancy and may serve as a potentially promising tool for the follow-up of various chronic diseases [8]. To our knowledge, this is the first study assessing life satisfaction in PE patients. We demonstrated an average result of “slightly satisfied” among our cohort. Mental disease, comorbidities and increased age were more frequently associated with life dissatisfaction, while trait and state anxiety were correlated with satisfaction with life. Only MH and GH were independent variables of SWLS results. Studies have previously show that life satisfaction is lower in patients with mental diseases [35], while anxiety has been found to be inversely correlated with life satisfaction among patients with other respiratory diseases [36]. Our results agree with the latter observations and provide insight into the psychological consequences of PE as far as life satisfaction is concerned.

PE is a potentially life-threatening state, mostly during the acute phase of the disease; PE patients commonly experience worrying symptoms such as dyspnea, hemoptysis and syncope [1]. The stress of hospitalization and the application of (invasive) treatment in PE patients may serve as potential stressors and lead to mood and psychological impairments [37]. We observed suboptimal state anxiety and borderline clinically significant trait anxiety in our cohort. MH score was an independent variable of trait anxiety. In the same context, others [38] have reported higher levels of anxiety in a small cohort of PE subjects. Arterial partial oxygen pressure (PaO_2_), disease severity and age influenced the levels of anxiety. The lower levels of anxiety experienced by our patients (when compared to Liu et al.) may be associated with lower age and more comorbidities in their cohort that may have led to increased anxiety.

QoL after PE has not been extensively studied in the literature. Impaired HRQoL among PE patients has been reported previously [6,39]. Mean SF-36 subscales among patients with PE are statistically significantly lower than in age- and sex-matched controls, as well as when compared to patients with severe/very severe COPD and congestive heart failure [39]. Our patients exhibited worse scores in almost all subscales when compared to the Greek general population norms. This is in accordance with data in the literature [6], although HRQoL in our cohort seems lower than previously reported by other groups [6,39]. One possible explanation is discrepancies in the time span between inclusion in the study and acute event, which was higher in previously published studies than in ours. As discussed later, disease duration is possibly associated with improved outcomes in QoL. Alternatively, the reduced HRQoL may reflect the effect of the COVID-19 pandemic, which may have affected the mental state of our sample.

Reduced disease duration was correlated with worse HRQoL, while patients with worse SF-36 subscale scores had significantly higher disease duration when compared to those with better HRQoL. Disease duration served as an independent variable for predictions of change in health. Our results may indicate that when patients are close to an event, they experience a decline in QoL that is followed by an improvement; our data strengthen those of other studies of VTE populations [6,38,39,40,41]. Lukas et al. [42] studied a mixed PE/DVT population and reported that increased time since the diagnosis was statistically significantly correlated with improved physical QoL. In the same context, others have demonstrated that the PF, SF and VTSF-36 subscales are higher (i.e., reflecting better QoL) in patients with longer duration since an event [6], although when QoL was assessed with other instruments (e.g., PEmb-Qol) this finding was not replicated.

In contrast to traditional perceptions, PE is now acknowledged as a chronic disease that in most patients requires sustained attention and close follow-up [43]. The mental and physical recovery of some patients may not reach completion, and studies have reported increased use of psychotropic drugs in VTE patients up to 5 years following an acute event [44]. Although some have reported that average QoL scores in PE patients improve and approach the normal values of healthy subjects within 1 year [45], others have demonstrated significant signs of post-traumatic syndrome > 9 months following diagnosis [12]. The aforementioned findings suggest that the long-term mental health of PE patients may be impaired and merits attention. Long-term interventions to support the mental health of this group of patients may have a beneficial health impact. Our results, in conjunction with those of others, may be used to design a structured follow-up program that addresses the mental health burden in this population. An early recognition may allow early intervention to enhance mental recovery and possibly improve the HRQoL of PE patients.

Age served as an independent variable for the prediction of some aspects of HRQoL (including PF and GH). In our cohort, older patients had lower SF-36 scores, reflecting impaired HRQoL. Our findings strengthen those of others reporting similar results [40]. In the same context, Kahn et al. [41] reported that age was predictive of SF-36 physical component scores in a cohort of patients with DVT. Older patients may experience more adverse events, have more complications during hospitalization and more frequently have comorbidities, which may collectively influence QoL.

Our study was performed during the COVID-19 pandemic. A high frequency of PE has been reported in COVID-19 patients, with some studies reporting increased severity and worse outcomes [46]. We observed that patients with PE associated with COVID-19 had lower change-in-health scores when compared to patients whose events were not COVID-19-related. The COVID-19 pandemic affects medical aspects of health as well as having social and economic effects. Restrictions and safety measures that were introduced during the COVID-19 pandemic have proven impactful on the mental health and QoL of patients suffering from various diseases [19,20,47,48], of the general population [49] and of frontline healthcare workers [50]. Our findings are consistent with previous studies and indicate the effects that COVID-19 may have on mental health. We must acknowledge that the strength of our findings is limited since only 4.4% of our cohort experienced COVID-19-related events. Due to the absence of previous assessments of HRQoL, anxiety and satisfaction, no definite conclusion can be drawn. In addition, one must acknowledge that the pandemic itself may have an impact on various aspects of mental health, impacting our results. Since COVID-19 may further complicate the mental health of patients with various chronic conditions [19,20], individuals with comorbidities may be more susceptible to mental health impairments.

This study is not without limitations. We acknowledge that we applied the psychometric questionnaires during the patients’ follow-up at the PE Outpatient Clinic of Larissa, Greece, and we did not perform longitudinal assessments; therefore, we cannot address potential differences in QoL, life satisfaction and anxiety over time. Additionally, we used convenience sampling and there is a potential selection bias. However, the characteristics of our sample suggest that our results may not lack external validity. We have included a small sample size. We acknowledge that our findings may have been affected by the decision to assess patients during the COVID-19 pandemic, which may have affected the mental burden of our patients. Unfortunately, we did not include a control group consisting of the patients prior to the COVID-19 pandemic, and therefore we cannot say whether our findings reflect the results of PE or the mental health burden of COVID-19. Additionally, we acknowledge that the majority of our patients presented comorbidities that may have implications for the findings of our study. However, we chose to include a real-world population in order to enhance the generalizability of our results.

In conclusion, we have demonstrated that patients with PE exhibit slight satisfaction with life, borderline anxiety and impaired HRQoL. Our results provide further insight into the mental consequences of PE. Although our study was performed during the pandemic, which may serve as a confounder, our results suggest that patients with PE exhibit long-term mental and QoL consequences, possibly due to their disease. Our data strengthen the concept that PE is a disease that requires a structured follow-up and sustained attention.

## Figures and Tables

**Table 1:** Sociodemographic characteristics of the study population.

Characteristic	Mean ± SD or %
Age (years)	62.5 ± 15.33
Gender	
Male	56.5%
Female	43.5%
Smoking status	
Non-smoker	45.7%
Ex-smoker	40.2%
Current smoker	14.1%
Comorbidities	
None	17.4%
≥1	82.6%
Comorbidities diagnosis	
Arterial hypertension	35.9%
Dyslipidemia	32.6%
Hashimoto thyroiditis	14.1%
Coronary artery disease	10.9%
Diabetes mellitus type IΙ	10.9%
Mental disease	8.7%
COVID-19	4.4%
Chronic Obstructive Pulmonary Disease	3.3%
Osteoporosis	2.2%
Obstructive sleep apnea hypopnea syndrome	1.1%
Idiopathic thrombocytosis	1.1%
Dementia	1.1%
Sarcoidosis	1.1%
Ulcerative colitis	1.1%
BMI	
Healthy weight/Overweight/Obesity	15.2%/40.2%/44.6%
Educational level	
Primary school	41.1%
3 years of high school	25.6%
6 years of high school	23.3%
university graduates	8.9%
Working status	
Unemployed/Retired/Currently working	15.2%/56.5%/27.2%
Marital status	
Married/In a relationship	78.3%
Single	9.8%
Widower	10.9%

**Table 2:** SF-36, STAI and SWLS results in our PE population compared to the Greek healthy population norms [25]. * *p* < 0.001 when compared to Greek norms, ^#^ *p* = 0.063 when compared to Greek norms. Abbreviations: SD, standard deviation; SE, standard error.

Parameter	Mean (±SD)	Median (±SE)	Mean (±SD) of the Greek Population [25]	Median of the Greek Population [25]
Physical functioning	57.77 (±25.76)	60.00 (±2.68)	80.76 (±25.62)	90 *
Physical role functioning	45.92 (±42.41)	41.65 (±1.42)	79.74 (±37.72)	100 *
Emotional role functioning	51.26 (±43.31)	6.63 (±4.51)	81.53 (±36.31)	100 *
Vitality	57.11 (±21.27)	60.00 (±2.21)	66.53 (±22.39)	70 *
Mental health	66.70 (±19.49)	68.00 (±2.03)	68.23 (±21.26)	72 ^#^
Social role functioning	61.95 (±31.49)	62.50 (±3.28)	82.05 (±28.12)	100 *
Bodily pain	72.44 (±28.40)	77.50 (±2.96)	72.98 (±31.66)	84 *
General health perceptions	54.02 (±18.79)	55.00 (±1.96)	67.46 (±23.54)	72 *
Change in health	58.42 (±30.62)	50.00 (±3.19)	-	-
STAIX1	37.05 (±11.17)	36.00 (±1.17)	-	-
STAIX2	39.80 (±10.47)	38.50 (±1.10)	-	-
SWLS	23.31 (±6.58)	25.00 (±0.69)	-	-

**Table 3:** Bivariate correlations of demographic parameters and SF-36 subscale, STAIX1, STAIX2 and SWLS scores. Abbreviations: PF, physical functioning; RP, physical role functioning; RE, emotional role functioning; VT, vitality; MH, mental health; SF, social role functioning; BP, bodily pain; GH, general health perceptions.

Parameters	SF-36 Subscales											
	PF	RP	RE	VT	MH	SF	BP	GH	Change in Health	STAIX1	STAIX2	SWLS
Age (years)	*p* < 0.001 r = −0.445	*p* = 0.003 r = −0.307	*p* > 0.05	*p* = 0.009 r = −0.270	*p* > 0.05	*p* < 0.001 r = −0.363	*p* > 0.05	*p* = 0.05 r = −0.293	*p* > 0.05	*p* > 0.05	*p* > 0.05	*p* > 0.05
Disease duration (years)	*p* > 0.05	*p* = 0.006 r = 0.292	*p* = 0.031 r = 0.229	*p* > 0.05	*p* > 0.05	*p* = 0.010 r = 0.270	*p* > 0.05	*p* > 0.05	*p* = 0.001 r = 0.347	*p* > 0.05	*p* > 0.05	*p* > 0.05
BMI (kg/m^2^)	*p* < 0.001 r = −0.515	*p* > 0.05	*p* > 0.05	*p* = 0.048 r = −0.281	*p* > 0.05	*p* > 0.05	*p* = 0.009 r = −0.368	*p* > 0.05	*p* > 0.05	*p* > 0.05	*p* > 0.05	*p* > 0.05

**Table 4:** Bivariate correlations between the SF-36 subscale, STAIX1, STAIX2 and SWLS scores. Abbreviations: PF, physical functioning; RP, physical role functioning; RE, emotional role functioning; VT, vitality; MH, mental health; SF, social role functioning; BP, bodily pain; GH, general health perceptions.

Parameters	SF-36 Subscales											
	PF	RP	RE	VT	MH	SF	BP	GH	Change in Health	STAIX1	STAIX2	SWLS
PF	-	*p* < 0.001 r = 0.553	*p* = 0.001 r = 0.336	*p* < 0.001 r = 0.530	*p* = 0.036 r = 0.219	*p* < 0.001 r = 0.515	*p* < 0.001 r = 0.481	*p* < 0.001 r = 0.380	*p* > 0.05	*p* = 0.016 r = −0.252	*p* = 0.010 r = −0.270	*p* = 0.017 r = 0.251
RP	*p* < 0.001 r = 0.553	-	*p* < 0.001 r = 0.595	*p* < 0.001 r = 0.444	*p* = 0.048 r = 0.207	*p* < 0.001 r = 0.603	*p* = 0.015 r = 0.252	*p* = 0.010 r = 0.268	*p* = 0.029 r = 0.228	*p* = 0.030 r = −0.228	*p* = 0.007 r = −0.281	*p* = 0.032 r = 0.229
	*p* = 0.001 r = 0.336	*p* < 0.001 r = 0.595	-	*p* < 0.001 r = 0.490	*p* < 0.001 r = 0.411	*p* < 0.001 r = 0.481	*p* = 0.024 r = 0.235	*p* < 0.001 r = 0.357	*p* = 0.035 r = 0.220	*p* < 0.001 r = −0.438	*p* < 0.001 r = −0.447	*p* < 0.001 r = 0.412
Anticoagulants (yes/No)	*p* < 0.001 r = 0.530	*p* < 0.001 r = 0.444	*p* < 0.001 r = 0.490	-	*p* < 0.001 r = 0.655	*p* < 0.001 r = 0.649	*p* < 0.001 r = 0.404	*p* < 0.001 r = 0.628	*p* = 0.003 r = 0.310	*p* < 0.001 r = −0.607	*p* < 0.001 r = −0.639	*p* < 0.001 r = 0.591
MH	*p* = 0.036 r = 0.219	*p* = 0.048 r = 0.207	*p* < 0.001 r = 0.411	*p* < 0.001 r = 0.655	-	*p* < 0.001 r = 0.450	*p* > 0.05	*p* < 0.001 r = 0.401	*p* = 0.028 r = 0.229	*p* < 0.001 r = −0.660	*p* < 0.001 r = −0.789	*p* < 0.001 r = 0.667
SF	*p* < 0.001 r = 0.515	*p* < 0.001 r = 0.603	*p* < 0.001 r = 0.481	*p* < 0.001 r = 0.649	*p* < 0.001 r = 0.450	-	*p* < 0.001 r = 0.383	*p* > 0.05	*p* > 0.05	*p* < 0.001 r = −0.379	*p* < 0.001 r = −0.429	*p* < 0.01 r = 0.431
BP	*p* < 0.001 r = 0.481	*p* = 0.015 r = 0.252	*p* = 0.024 r = 0.235	*p* < 0.001 r = 0.404	*p* > 0.05	*p* < 0.001 r = 0.383	-	*p* = 0.003 r = 0.302	*p* > 0.05	*p* > 0.05	*p* > 0.05	*p* = 0.009 r = 0.275
GH	*p* < 0.001 r = 0.380	*p* = 0.010 r = 0.268	*p* < 0.001 r = 0.357	*p* < 0.001 r = 0.628	*p* < 0.001 r = 0.401	*p* > 0.05	*p* = 0.003 r = 0.302	-	*p* > 0.05	*p* < 0.001 r = −0.541	*p* < 0.001 r = −0.534	*p* < 0.001 r = 0.572
Change in health	*p* > 0.05	*p* = 0.029 r = 0.228	*p* = 0.035 r = 0.220	*p* = 0.003 r = 0.310	*p* = 0.028 r = 0.229	*p* > 0.05	*p* > 0.05	*p* > 0.05	-	*p* > 0.05	*p* > 0.05	*p* > 0.05
STAIX1	*p* = 0.016 r = −0.252	*p* = 0.030 r = −0.228	*p* < 0.001 r = −0.438	*p* < 0.001 r = −0.607	*p* < 0.001 r = −0.660	*p* < 0.001 r = −0.379	*p* > 0.05	*p* < 0.001 r = −0.541	*p* > 0.05	-	*p* < 0.001 r = 0.761	*p* < 0.001 r = −0.569
STAIX2	*p* = 0.010 r = −0.270	*p* = 0.007 r = −0.281	*p* < 0.001 r = −0.447	*p* < 0.001 r = −0.639	*p* < 0.001 r = −0.789	*p* < 0.001 r = −0.429	*p* > 0.05	*p* < 0.001 r = −0.534	*p* > 0.05	*p* < 0.001 r = 0.761	-	*p* < 0.001 r = −0.669
SWLS	*p* = 0.017 r = 0.251	*p* = 0.032 r = 0.229	*p* < 0.001 r = 0.412	*p* < 0.001 r = 0.591	*p* < 0.001 r = 0.667	*p* < 0.01 r = 0.431	*p* = 0.009 r = 0.275	*p* < 0.001 r = 0.572	*p* > 0.05	*p* < 0.001 r = −0.569	*p* < 0.001 r = −0.669	-

**Table 5:** Multivariate linear regression model for possible independent predictors of STAIX1, STAIX2 and SWLS scores. Abbreviations: PF, physical functioning; RP, physical role functioning; RE, emotional role functioning; VT, vitality; MH, mental health; SF, social functioning; BP, bodily pain; GH, general health perceptions.

	SWLS		STAIX1		STAIX2	
	β	*p*	β	*p*	β	*p*
MH	0.113	**0.015**	−0.042	0.579	−0.242	**<0.001**
GH	0.105	**0.003**	−0.077	0.193	−0.071	0.108
SF	0.011	0.654	−0.009	0.802	0.034	0.226
BP	0.027	0.189	-	-	-	-
RP	−0.003	0.876	0.033	0.233	−0.020	0.335
RE	0.011	0.491	−0.034	0.188	−0.016	0.410
PF	−0.022	0.404	0.022	0.591	−0.003	0.922
VT	−0.002	0.971	−0.089	0.199	0.010	0.851
STAIX1	−0.014	0.851	-	-	0.312	**<0.001**
STAIX2	−0.095	0.312	0.549	**<0.001**	-	-
SWLS	-	-	0.13	0.942	−0.145	0.293
R squared	0.581		0.635		0.756	

## Data Availability

The data are not publicly available due to data privacy regulations.

## Supplementary Materials

The following are available online at https://www.mdpi.com/article/10.3390/jpm14040393/s1, Figure S1: Comparison of Physical Functioning levels in males versus females, in patients with or without dyspnea at presentation or hemoptysis at presentation and comparison of Bodily pain subscale in patients with versus without history of recurrence, in patients with versus without current use of anticoagulants and patients with versus without history of COVID-19 related Pulmonary embolism; Figure S2: Comparison of SWLS score, Social functioning subscale and General Health subscale in patients with at least one comorbidity versus those without; Figure S3: Comparison of STAI X1, STAIX2 and SWLS levels as well as Vitality, Mental Health and General Health subscale in patients with and without mental disease; Figure S4: Comparison of Role emotional, Mental Health and General Health subscales as well as STAIX1 levels and SWLS levels in patients with and without diabetes mellitus; Figure S5: Comparison of Physical Functioning and Bodily Pain subscale in patients with and without thyroid disease. Comparison of Physical Functioning, Role emotional, Social Functioning and Bodily Pain subscale in patients with and without hereditary thrombophilia. Table S1: Cronbach alpha value of the questionnaires studied; Table S2: Patient symptoms at diagnosis.
