# Supplementary material for: Investigation of Health-Related Quality of Life, Anxiety and Satisfaction in Patients with Pulmonary Embolism
Source: J Pers Med. 2024 Apr 8;14(4):393. doi: 10.3390/jpm14040393 (PMC11051348; doi:10.3390/jpm14040393)
Supplement: Supplementary file 1 [file jpm-14-00393-s001.zip › jpm-2937009-supplementary.pdf]

Supplementary Figure S1

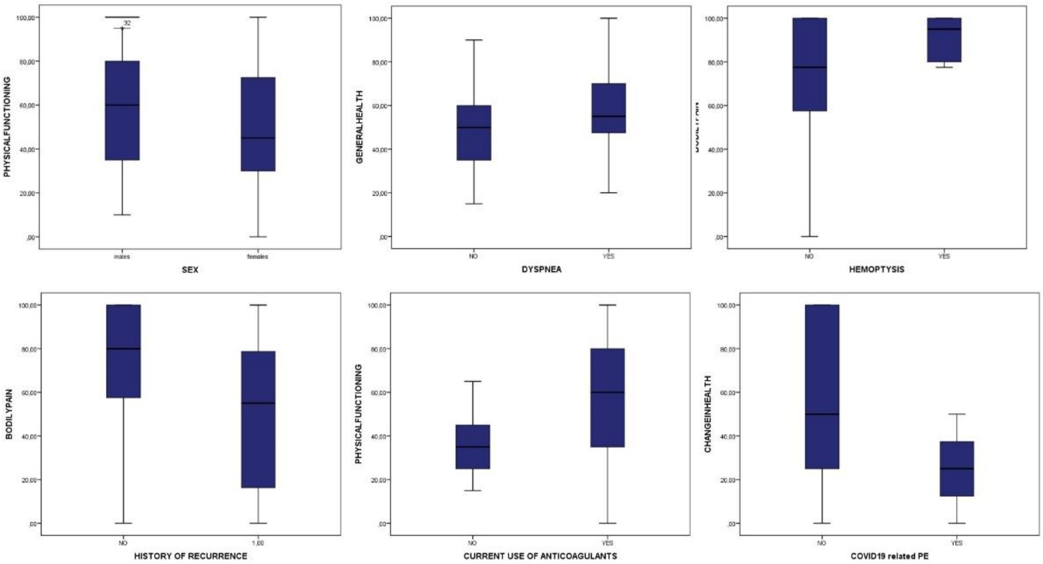

Supplementary Figure S2

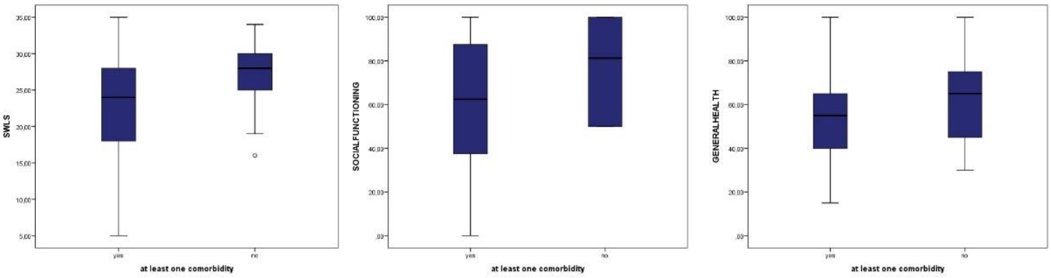

Supplementary Figure S3

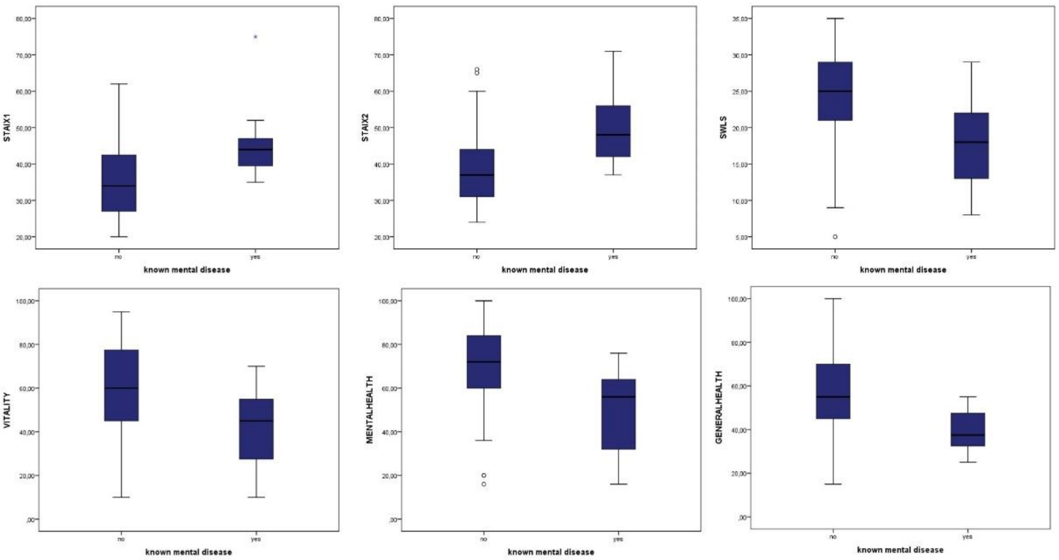

Supplementary Figure S4

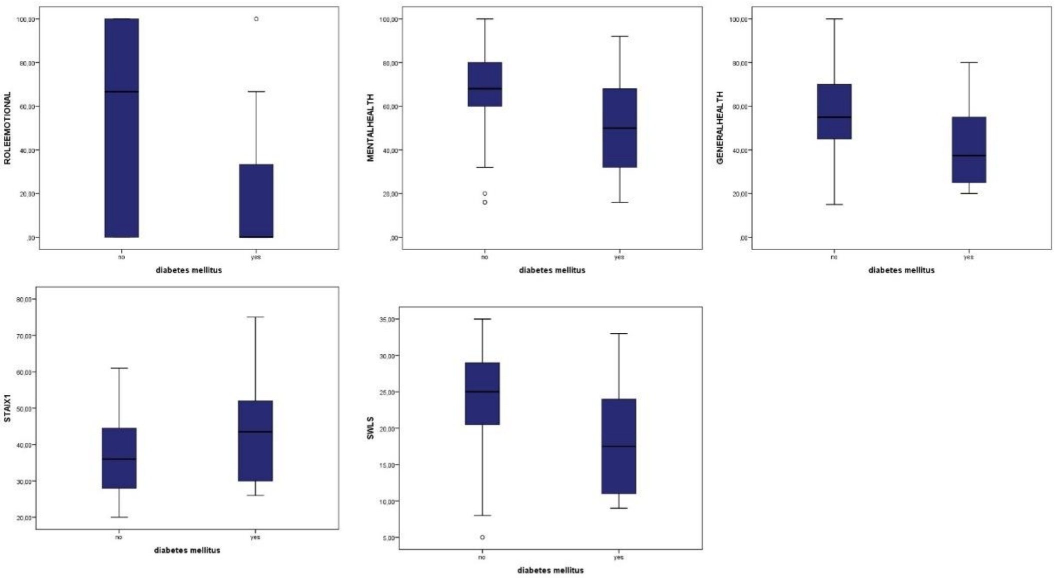

Supplementary Figure S5

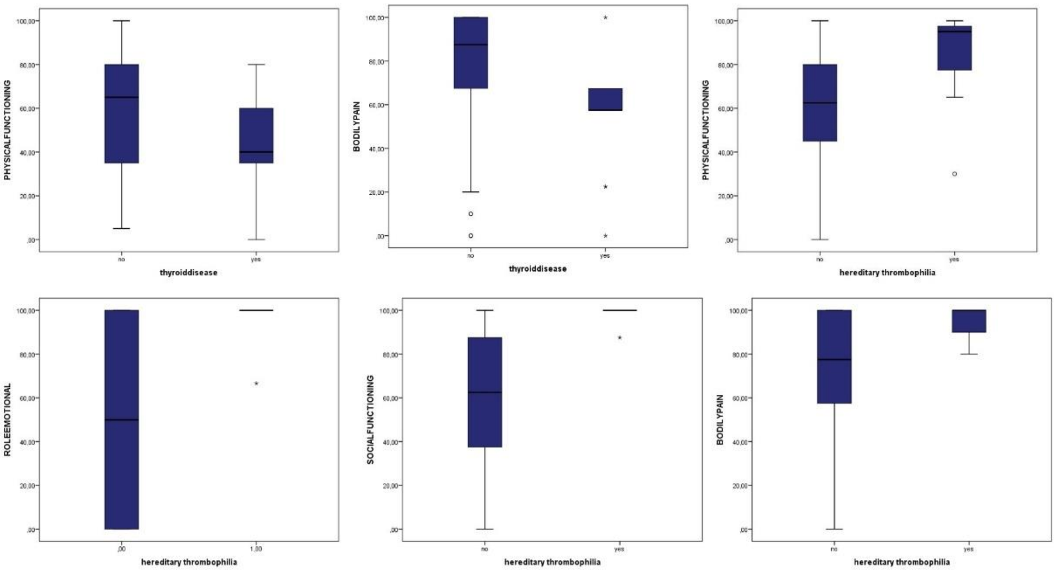

Supplementary Table S1

Dimension	Cronbach's alpha
SF36 Physical functioning	0.92
Social role functioning	0.80
Role physical functioning	0.90
Emotional role	0.85
Vitality	0.91
Mental health	0.89
Body pain	0.86
General health perceptions	0.90
STAIX1	0.85
STAIX2	0.91
SWLS	0.86

Supplementary Table S2. Patient symptoms at diagnosis.

Symptomσ at PE diagnosis	Percentage (%)
Dyspnea	52,2 %
Cough	8,7%
Hemoptysis	6,5%
Chest pain	32,6%
Other	51,1%
